# A New Method for Precision Cold Neutron Polarimetry Using a ^3^He Spin Filter

**DOI:** 10.6028/jres.110.044

**Published:** 2005-06-01

**Authors:** F. E. Wietfeldt, T. R. Gentile

**Affiliations:** Tulane University, New Orleans, LA 70118; National Institute of Standards and Technology, Gaithersburg, MD 20899

**Keywords:** cold neutron, polarimetry, ^3^He spin filter

## Abstract

We present a new method for precision measurement of the capture flux polarization of a polychromatic (white), continuous cold neutron beam, polarized by a ^3^He spin filter. This method allows an *in situ* measurement and does not require knowledge of the neutron beam wavelength distribution. We show that a polarimetry precision of 0.1 % is possible.

## 1. Introduction and Discussion

The ^3^He nucleus absorbs neutrons through the reaction ^3^He + *n* → ^3^H + *p* + 764 keV. The cross section is very large (*σ*_th_ = 10666 b) when the spins are antiparallel and very small when the spins are aligned. This strong spin dependence makes polarized ^3^He an ideal spin filter for producing spin-polarized epithermal, thermal, and cold neutron beams [1,2]. A ^3^He neutron spin filter can also be used as a neutron polarization analyzer [3–5]. There are two standard methods for polarizing the ^3^He: spin exchange collisions with an optically-pumped Rb vapor [1], and metastability-exchange in excited ^3^He [6].

Highly polarized (> 90 %) cold neutron beams are used for measuring the parity-violating beta asymmetry (*A*) and neutrino asymmetry (*B*) correlation coefficients in neutron beta decay [7–9]. Because neutron decay is such a simple system, these coefficients are directly related to the weak vector and axial vector coupling constants *g*_V_ and *g*_A_. Precision measurements of these parameters can test the unitarity of the Cabibbo-Kobayashi-Maskawa (CKM) matrix, provide limits on weak scalar and tensor currents, right-handed currents, conserved vector current (CVC) violation and second-class currents, and other possible new physics beyond the Standard Model of particle physics. These experiments have or will be carried out at the NIST Center for Neutron Research, the Institut Laue-Langevin in Grenoble, France, and the new Spallation Neutron Source in Oak Ridge, Tennessee. The parameters *A* and *B* are currently known to a precision of about 1 % [10], and the next generation experiments plan to push the precision down to the 0.1 % level. To accomplish this, cold neutron polarimetry at the most intense available neutron beams must also reach a reliable precision of 0.1 %. Neutron polarimetry below the 1 % level of precision has been a notoriously difficult problem for these experiments. The objective of this paper is to outline a method by which the polarization of a polychromatic (white) cold neutron beam can be measured *in situ* at a level of 0.1 % precision.

Consider a ^3^He cell that contains atomic densities $N_{3}^{+}$ and $N_{3}^{-}$ of the two spin states $\pm \frac{1}{2}$, with $N_{3}^{+}>N_{3}^{-}$. The total atomic density is $N_{3}=N_{3}^{+}+N_{3}^{-}$ and we define the ^3^He polarization $P_{3}$to be:

$$P_{3}\equiv \frac{N_{3}^{+}-N_{3}^{-}}{N_{3}}$$ (1)

The neutron absorption cross section *σ* is inversely proportional to neutron velocity, and therefore proportional to the neutron’s deBroglie wavelength *λ*. Therefore it has a wavelength dependence:

$$\sigma (\lambda )=\left(\frac{\sigma_{\text{th}}}{\lambda_{\text{th}}}\right)\lambda$$ (2)

where *σ*_th_ = 5333 b is the absorption cross section for unpolarized neutrons at the canonical thermal wavelength *λ*_th_ = 0.180 nm. The neutron transmission of the cell for neutron spin $\pm \frac{1}{2}$ is then:

$$T^{\pm }(\lambda ,P_{3})=T_{E}(\lambda )\exp\left\{-2\left(\frac{\sigma_{\text{th}}}{\lambda_{\text{th}}}\right)\lambda N_{3}^{\mp }x\right\}$$ (3)

where *T*_E_(*λ*) is the transmission of the empty (evacuated) cell and *x* is the cell length. For an unpolarized monochromatic (single wavelength) incident neutron beam, the neutron polarization exiting the cell is:

$$P_{n}=\frac{T^{+}-T^{-}}{T^{+}+T^{-}}=\frac{T_{E}(\lambda )\exp\{-\chi \lambda \}\sinh\{\chi \lambda P_{3}\}}{T_{E}(\lambda )\exp\{-\chi \lambda \}\cosh\{\chi \lambda P_{3}\}}=\tanh\{\chi \lambda P_{3}\}$$ (4)

where

$$\chi =\left(\frac{\sigma_{\text{th}}}{\lambda_{\text{th}}}\right)N_{3}x$$ (5)

is a fixed property of the cell. Note also that the ratio of the total cell transmission with a polarized/unpolarized cell is:

$$\frac{T(\lambda ,P_{3})}{T(\lambda ,P_{3}=0)}=\cosh\{\chi \lambda P_{3}\}$$ (6)

so with a *monochromatic* beam, the neutron polarization exiting the cell is determined by transmission measurements using the same cell polarized and unpolarized [1]:

$$P_{n}=\sqrt{1-{\left(\frac{T(\lambda ,P_{3}=0)}{T(\lambda ,P_{3})}\right)}^{2}}.$$ (7)

At a pulsed (e.g., spallation) neutron source the neutron wavelength in the cell is known by time-of-flight from the target relative to the pulse, so Eq. (7) can be used for precise polarimetry using a ^3^He polarizer. However at present, and for the foreseeable future, the most intense cold neutron beams are at continuous, reactor-based, neutron sources. A continuous source is characterized by a time-independent, polychromatic neutron wavelength spectrum, so Eq. (7) can be used only with an upstream beam chopper or wavelength selector, which significantly reduces the integrated neutron fluence. Use of a separate ^3^He analyzer cell at a continuous source presents other technical problems. Hence for a polychromatic neutron beam we must integrate over wavelength [compare to Eq. (4)]:

$$P_{n}=\frac{\int n(\lambda )T_{E}(\lambda )\exp\{-\chi \lambda \}\sinh\{\chi \lambda P_{3}\}d\lambda }{\int n(\lambda )T_{E}(\lambda )\exp\{-\chi \lambda \}\cosh\{\chi \lambda P_{3}\}d\lambda }.$$ (8)

Here *n*(*λ*) is the beam wavelength distribution. This expression cannot be simplified because the integrands don’t cancel. For polarized neutron decay experiments the more important quantity is the “capture flux” neutron polarization which is weighted by *λ* to reflect the *λ* weighting of neutron decay probability within the experimental detector:

$$P_{n}^{C}=\frac{\int n(\lambda )\lambda T_{E}(\lambda )\exp\{-\chi \lambda \}\sinh\{\chi \lambda P_{3}\}d\lambda }{\int n(\lambda )\lambda T_{E}(\lambda )\exp\{-\chi \lambda \}\cosh\{\chi \lambda P_{3}\}d\lambda }.$$ (9)

This is the quantity that must be measured precisely. An experimental evaluation of the integrals in Eq. (9) is difficult, and an overall determination of $P_{n}^{C}$ to a precision of 0.1 % or better is problematic at a continuous neutron source using existing methods.

**Fig. 1:**
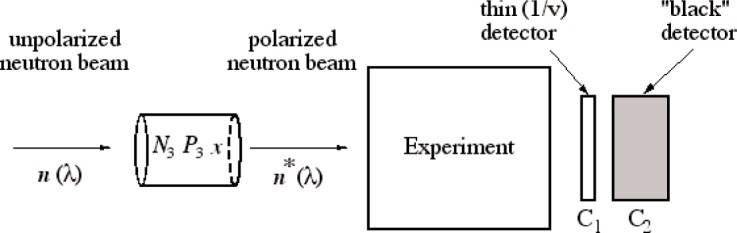
Schematic representation of the proposed method.

We propose a novel approach to precise neutron polarimetry on a polychromatic beam that promises to achieve a precision of less than 0.1 %. The basic idea is shown in Fig. 1. A polarized ^3^He cell is used to produce a polarized neutron beam with wavelength distribution n*(*λ*) and capture flux polarization $P_{n}^{C}$ Eq. (9). The beam passes through the experiment, and then through two neutron detectors: a “thin” detector *C*_1_ and a “black” detector *C*_2_. The thin detector would be a foil of a strong neutron absorber, such as ^6^Li or ^10^B, thin enough that the neutron transmission loss through the foil is negligible. Neutrons are detected by counting the reaction products. Such detectors have been used in previous neutron decay experiments for absolute flux measurements [11] and they work very well. A thin detector of this type has an efficiency that is precisely proportional to neutron wavelength (the 1/*v* law). The black detector contains a thick absorber so that practically all incident neutrons are absorbed. A commercially-available ^3^He ionization chamber would be suitable for this.

The measured count rate in the thin detector *C*_1_ is:

$$\begin{array}{l} N_{1}=\int \varepsilon_{1}n*(\lambda )\lambda d\lambda \\ =\varepsilon_{1}\int n(\lambda )\lambda T_{E}(\lambda )\exp\{-\chi \lambda \}\cosh\{\chi \lambda P_{3}\}d\lambda \end{array}$$ (10)

and the measured count rate in the black detector *C*_2_ is:

$$\begin{array}{l} N_{2}=\int \varepsilon_{2}n*(\lambda )d\lambda \\ =\varepsilon_{2}\int n(\lambda )T_{E}(\lambda )\exp\{-\chi \lambda \}\cosh\{\chi \lambda P_{3}\}d\lambda \end{array}$$ (11)

where *ε*_1_ and *ε*_2_ are wavelength-independent efficiency constants. Now assume that *P*_3_ is varied and that we can measure:

$$\left|\frac{dN_{2}}{dP_{3}}\right|=\varepsilon_{2}\int n(\lambda )T_{E}(\lambda )\exp\{-\chi \lambda \}\lambda \sinh\{\chi \lambda P_{3}\}d\lambda .$$ (12)

We find that, by combining Eqs. (9), (10), and (12), we have:

$$P_{n}^{C}=\left(\frac{\varepsilon_{1}}{\varepsilon_{2}\chi }\right)\frac{\left|\frac{dN_{2}}{dP_{3}}\right|}{N_{1}}.$$ (13)

Thus we have found a way to relate the precise capture flux polarization $P_{n}^{C}$ of a polychromatic beam to neutron count rate measurements that are made *in situ* during the experiment. We emphasize that Eq. (13) holds for any neutron wavelength distribution, monochromatic or polychromatic. With this method there is no need to account for or measure the wavelength distribution of the beam. Note that *ε*_1_, *ε*_2_, and *χ* do not depend on wavelength or ^3^He polarization. They can be determined precisely by a separate calibration measurement on a monochromatic beam. The most challenging part of this scheme will be a precise measurement of d*N*_2_/d*P*_3_. All other aspects should not present difficulty.

**Fig. 2:**
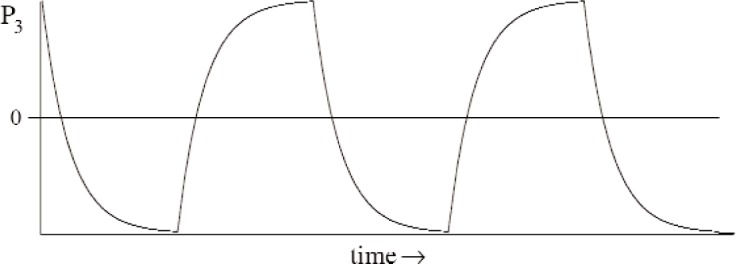
The variation of *P*_3_ with time if the laser polarization direction is reversed periodically.

It is best to vary *P*_3_ periodically so that data obtained over many cycles can be combined to reduce the uncertainty. For example, we could rotate the quarter-wave plate on the laser source twice per day to reverse the laser polarization. This would produce an exponential sawtooth in *P*_3_, as shown in Fig. 2. It would also serve as an additional spin-flip for the experiment, which is useful for investigating systematic effects.

We find d*N*_2_/d*P*_3_ by combining the measured d*N*_2_/d*t* with the known function d*P*_3_/d*t*. Now *N*_2_ will have time dependence from the variation of *P*_3_, and also from the beam intensity which is not constant. We can measure the beam intensity independently using a black beam monitor in an upstream part of the beam away from the experimental beam. Neutron absorption rates in both the beam monitor and the black detector *C*_2_ will be about 10^9^ s^−1^ so this can be done with very high statistical precision. The limit of our technique will be the precision on d*P*_3_/d*t*.

The polarized ^3^He will produce a large NMR signal so a precise relative determination of d*P*_3_/d*t* can be made using NMR. We will also require an absolute calibration. The standard technique of absolute comparison to a water cell NMR signal will not be precise enough for this purpose. Instead we propose to conduct a separate measurement on a monochromatic neutron beam, where we can compare the neutron beam polarization determined simultaneously from Eqs. (7) and (13) to provide an absolute calibration of the NMR signal to a precision of within 0.1 %.
